# Glycogen synthase kinase‐3β inhibition decreases inflammation and relieves cancer induced bone pain via reducing Drp1‐mediated mitochondrial damage

**DOI:** 10.1111/jcmm.17432

**Published:** 2022-06-10

**Authors:** He‐Yu Yang, Feng Zhang, Meng‐Lin Cheng, Ji Wu, Min Xie, Liang‐Zhu Yu, Ling Liu, Jun Xiong, Hai‐Li Zhu

**Affiliations:** ^1^ School of Pharmacy Hubei University of Science and Technology Xianning China; ^2^ School of Basic Medical Sciences Hubei University of Science and Technology Xianning China; ^3^ Clinical College of Youjiang Medical University for Nationalities Baise Guangxi China

**Keywords:** cancer‐induced bone pain, dynamic‐related protein 1, glycogen synthase kinase‐3β, mitochondria, spinal inflammation

## Abstract

Bone is the preferential site of metastasis for breast cancer. Invasion of cancer cells induces the destruction of bone tissue and damnification of peripheral nerves and consequently induced central sensitization which contributes to severe pain. Herein, cancer induced bone pain (CIBP) rats exhibited destruction of tibia, mechanical allodynia and spinal inflammation. Inflammatory response mainly mediated by astrocyte and microglia in central nervous system. Our immunofluorescence analysis revealed activation of spinal astrocytes and microglia in CIBP rats. Transmission electron microscopy (TEM) observations of mitochondrial outer membrane disruption and cristae damage in spinal mitochondria of CIBP rats. Proteomics analysis identified abnormal expression of proteins related to mitochondrial organization and function. Intrathecally, injection of GSK‐3β activity inhibitor TDZD‐8 significantly attenuated Drp1‐mediated mitochondrial fission and recovered mitochondrial function. Inhibition of GSK‐3β activity also suppressed NLRP3 inflammasome cascade and consequently decreased mechanical pain sensitivity of CIBP rats. For cell research, TDZD‐8 treatment significantly reversed TNF‐α induced mitochondrial membrane potential (MMP) deficiency and high mitochondrial reactive oxygen species level. Taken together, GSK‐3β inhibition by TDZD‐8 decreases spinal inflammation and relieves cancer induced bone pain via reducing Drp1‐mediated mitochondrial damage.

## 1 INTRODUCTION

In 2020, there is 19.29 million new cancer cases worldwide, and 4.57 million new cancers occurred in China, which far exceeds other countries in the world.^1^ Breast cancer is the most frequent malignancy in women and is prone to bone metastasis.^2^ The invasion of bone by tumour cells in bone marrow cavity destructs bone tissue, impairs peripheral nerves and consequently induces noxious signals.^3^, ^4^ These noxious signals can sensitize the central nervous system and contribute to pain. Cancer induced bone pain (CIBP) is characterized as a moderate‐to‐severe pain, combining of background, spontaneous and incident pain, mixing with inflammatory, neuropathic pain and tumour‐specific mechanisms.^5^ World Health Organization and International Association for the Study Pain identified that CIBP is a global public health problem.^6^ Due to insufficient understanding of CIBP mechanism and limitations of existing clinical treatment, about 50% of patients with CIBP have not been effectively controlled, resulting in a serious decline in the quality of patients.^7^ Therefore, research on the mechanisms of CIBP and development on related analgesics are major issues which needs to be resolved urgently.

Noxious stimuli convert to noxious signals and conduct from periphery to the spinal cord, where the incoming messages are integrated, and finally transmit to many parts of the brain.^8^ The mechanisms of CIBP include changes in both peripheral and central nervous system. Depolarization of peripheral nerve terminals and loss or preservation of primary afferent fibres could be a consequence of allodynia and hyperalgesia.^9^ For central nervous system, especially in spinal cord, increased synaptic excitability and plasticity, enlarged receptive field areas, enhanced responses to evoked stimuli, activated glial cells and suppressed opioid system are involved in the central mechanism of pain induction.^10^ Activation of glial cells leads to reactive gliosis and consequently induces the release of several key pro‐inflammatory cytokines, enhancement of synapse excitability and plasticity and increase in pain hypersensitivity.^11^ Microglia and astrocytes are the most abundant glial cells in spinal cord. Under physiological conditions, microglia phagocytize infected or damaged cells to protect neurons.^12^ Astrocytes take up excitatory transmitter glutamate to modulate the external environment of neurons.^13^ During acute injury and chronic neurological disease process, microglia and astrocytes convert are became reactive state.^14^ Microglia in response to injury within minutes and undergoes rapid proliferation. The soma is hypertrophied and processes are retracted.^15^ In contrast, astrocytes undergo hyperplasia and hypertrophy in several days after injury and much longer‐lasting. Astrocytic cell body is enlarged and processes are thickened.^16^ Pro‐inflammatory mediators release from activated microglia and astrocytes activates intracellular inflammatory signalling.^17^

Mitochondria mainly functions on adenosine‐triphosphate (ATP) production and reactive oxygen species (ROS) generation.^18^ Mitochondrial dysfunction in glial cells is closely related to the pathological state, such as brain disorders and injury. Dysregulation of biological processes in mitochondria and mitochondrial dynamics in astrocytes, such as mitochondrial trafficking and disbalance of fission and fusion, are contribute to neuropathic mechanism.^19^, ^20^, ^21^ Moreover, mitochondrial perturbation and components release from mitochondria promote cytokines generation, consequently induce neuroinflammation.^22^ Our research also proved that the activation of astrocytes is accompanied by the increased mitochondrial fission in the rat model of bone cancer pain.^23^, ^24^

Coupled with high metabolic requirements, mitochondrial morphology undergoes fission and fusion.^25^ Fission products smaller and more discontinuous mitochondria, following with decreased ATP production and mitochondrial decoupling.^26^ Mitochondrial dysfunction with impaired ATP production and increased ROS content are related to pain induction.^27^ Mitochondrial fission is strictly controlled by GTPase dynamin‐related protein1 (DRP1). Over‐activated Drp1 increased mitochondrial fragmentation and disrupted balance of mitochondrial dynamics.^28^ Drp1 can be phosphorylated by glycogen synthase kinase 3β (GSK‐3β) which in turn affects mitochondrial morphology and fission status.^29^ GSK‐3β activation was found in spinal nerve ligation (SNL) animal and closely related to abnormal nociceptive behaviour; inhibition of GSK‐3β activity by antagonist can alleviated neuropathic pain.^30^ In the current study, we proposed that GSK‐3β inhibition by TDZD‐8 reduced Drp1 mediated mitochondrial fission to decrease inflammation reaction and relieve cancer induced bone pain.

## 2 MATERIALS AND METHODS

### 2.1 Cell preparation and treatment

MRMT‐1 rat breast cancer cells were cultured in RPMI1640 (Gibco) containing 10% foetal bovine serum, 50 U/ml penicillin and 50 μg/ml streptomycin at 37°C with 5% carbon dioxide. C6 glial cells were cultured in DMEM (Gibco) with 10% foetal bovine serum, supplement with 50 U/ml penicillin and 50 μg/ml streptomycin. For treatment, C6 cells were induced with 5 ng/μl TNF‐α for 4 h combined with 0 or 1 μM TDZD‐8 and mdivi‐1 treated for 24 h. After treatment, cells were digested by trypsin (Gibco) and collected for Western blot analysis. TDZD‐8 and mdivi‐1 (Selleck Chemicals) were dissolved in dimethyl sulfoxide (DMSO) (Beyotime Bio‐tech.) and diluted to different concentrations.

### 2.2 Experimental animals and TDZD‐8 treatment

Sprague–Dawley (SD) rats weighing 180–200 g were purchased from the Hubei Experimental Animal Center (China). They were housed in standard house maintained at 22 ± 1°C, kept a 12 h light/12 h dark cycle, allowed free access to water and food pellets. All experimental procedures in this study in accordance with local and international guidelines for the ethical use of animals and all effort was made to minimize the number and pain. Rats were intrathecally injected 10 μl TDZD‐8 (1 mg/kg) using a 25 μl microsyringe inserting between the L5 ~ L6 vertebrae. TDZD‐8 is dissolved in DMSO and diluted with 0.9% sodium chloride (v/v = 1:1) before using. Vehicles were injected with same volume (10 μl) of DMSO and 0.9% NaCl.

### 2.3 Cancer induced bone pain rat model establishment

MRMT‐1 cells were used to establish the model of CIBP and according to the previously research.^31^ Briefly, Rats were deeply anaesthetised with pentobarbital sodium (50 mg/kg, intraperitoneal injection). The left leg was disinfected with 7% iodine and 75% (v/v) ethanol, exposed the upper half of the tibia, and the suspension of 5.0 × 10^5^ MRMT‐1 cells was slowly injected into the intramedullary cavity of the left tibia. The equal volume of Hank's solution was injected into the same position in the sham groups. The syringe was stayed at the injection site for a minute to prevent the leakage of the tumour cells or solution. After the syringe was removed, the injection site was sealed with bone wax and finally sew it up.

### 2.4 Bone tissue testing with radiology and Haematoxylin and Eosin (H&E) staining

After cells inoculation for 14 days, rats were completely anaesthetised. Rapid transcardial perfusion of 0.9% NaCl perfusate containing heparin was carried out and subsequently switched perfusate to 4% polyformaldehyde. After perfusion, the legs were removed and preserved in 10% neutral buffer formalin. For roentgenography of tibia, the legs were placed on the X‐ray film and exposed to the X‐ray source. For H&E staining, the left tibial was decalcified using 10% EDTA for 7 days, embedded in paraffin, sliced to 4 μm thick (Leica RM2165, Leica Microsystems). Slides were baked, dewaxed, soaked, stained with H&E and observed under microscope (Olympus IX73, Olympus).

### 2.5 Pain Behaviour test

The 50% paw withdrawal threshold (PWT) was determined by Chaplan's up‐down method.^32^ The rats were placed in a transparent plastic box with 5 × 5 cm wire mesh grid floor and allowed to habituate for 30 min. Then, the von Frey filaments (0.4–26 g; Stoelting) were used to stimulate the left hind paw. The fibre was bended with maintaining 3 ~ 5 s, adjacent stimulation between 2 min and a brisk withdrawal was considered as a positive response. When a positive response occurred, the von Frey filament with the next lower force was used, conversely, the filament with the next higher force was used. Finally, the pattern of positive and negative withdrawal response was converted to 50% PWT.

### 2.6 Morphological detection of spinal cord

Rats were deeply anaesthetised and perfused with PBS followed by 4% paraformaldehyde. Spinal cords were removed and collected for paraffin embedding, the tissues were cut into 4‐μm sections using a microtome. Then, the sections were stained using standard H&E methods and Nissl dying (cresyl violet). Images were captured by microscopy (Olympus IX73, Olympus).

### 2.7 Spinal proteomics using Liquid chromatography–tandem mass spectrometry (LS/MS–MS)

Two frozen lumbar spinal cord were triturated with liquid nitrogen, lysed by sonication, extracted with phenolic extraction buffer (10 mM dithiothreitol, 1% protease inhibitors, and 2 mM EDTA), centrifugated at 5500 *g* for 10 min at 4°C. Supernatants were precipitated with 0.1 M ammonium acetate/methanol overnight. The precipitations were dissolved in 8 M. Protein concentrations were measured using Bradford assay. The samples were digested with trypsin at a mass ratio of 1:50 (trypsin: protein) overnight at 37°C. The digested peptides were freeze‐dried under vacuum and dissolved into mobile phase A (0.1% formic acid and 0.2% acetonitrile). Agilent 300 Extend C18 column (4.6 × 250 mm, 5 μm 110 Å) and UPLC system were used to fractionate each sample into 60 fractions with increasing mobile phase B (20 mM formic acid in acetonitrile). The peptides were separated by ultra‐performance liquid system, injected into an NSI ion source for ionization and analysed by Q Exactive HF mass spectrometry. The ion source voltage was set at 2.0 kV, and the peptide parent ion and its secondary fragments were detected and analysed using high resolution Orbitrap in Q Exactive HF. The resulting MS spectra were acquired across the mass range of 350–1200 m/z in high resolution mode (70,000) and the accumulation time is 250 ms per spectrum. A maximum of 40 precursors per cycle with a minimum accumulation time of 100 ms for each precursor and dynamic exclusion for 20 s were chosen for fragmentation from each MS spectrum. Tandem mass spectra were recorded in high sensitivity mode (resolution = 17,500) using rolling collision energy. Complete scanning information was matched with various database using analysis software PEAKS. All identified protein sequences were derived from UniProt Database.

### 2.8 Transmission electron microscopy (TEM)

The lumber spinal cord was isolated, cut into 1 mm^3^ cubes, fixed in 2.5% glutaraldehyde, post‐fixed with 1% osmium tetroxide, stained with 2% uranium acetate saturated alcohol solution avoid light and 2.6% lead citrate avoid CO_2_. The cuprum grids were observed under TEM (HT7800, HITACHI) and take images.

### 2.9 Western blot analysis

After 12 h TDZD‐8 and vehicle treatment, rats were anaesthetised and sacrificed by decapitation. Lumber spinal cord were collected and homogenized in RIPA lysis buffer containing 1% protease inhibitors (Sigma Aldrich). After centrifugation at 12,000 g, 4°C for 20 min, the supernatant was used for Western blotting analysis. Protein concentration was quantified via BCA analysis kit (Beyotime Bio‐tech). Protein lysates were separated on 10% SDS‐PAGE gels and transferred to 0.22 μm PVDF membranes. Membranes were subsequently incubated with specific primary antibodies at 4°C overnight. The following primary antibodies were used: anti‐pTyr216‐GSK3β (AP0261), anti‐GSK‐3β (A6164), anti‐pSer616‐Drp1 (AP0849), anti‐Drp1(A2586), anti‐NLRP3 (A5652), anti‐ASC (A1170), anti‐caspase‐1 (A0964), anti‐IL‐1β (A1112) and anti‐β‐actin (AC026) were from ABclonal Technology; anti‐cleaved IL‐1β (AF4006) was from Affinity Biosciences. HRP‐conjugated secondary antibodies (ABclonal Technology) were used to visualize the primary antibodies. iBright 1500 imaging system (Invitrogen) was applied to detect immunoreactive bands. The grey value of bands was analysed by ImageJ software.

### 2.10 Immunofluorescene

The spinal cord sections were dewaxed, underwent antigen repair, treated with 3% hydrogen peroxide for 10 min, washed with 1% PBS for 3 times, sealed with immunostaining blocking solution for 1 h, incubated with primary antibody overnight at 4°C and fluorescent secondary antibody at room temperature for 1 h and observed under fluorescence microscope (Olympus FV3000, Olympus). Primary antibodies anti‐Iba1 (A12391), anti‐GFAP (A0237), anti‐pTyr216‐GSK3β (AP0261), anti‐GSK‐3β (A6164), anti‐pSer616‐Drp1 (AP0849), anti‐Drp1 (A2586), anti‐NLRP3 (A5652), anti‐caspase‐1 (A0964), were from ABclonal Technology. Anti‐cleaved IL‐1β (AF4006) was from Affinity Biosciences. The intensity was analysed by ImageJ software.

### 2.11 Immunoprecipitation

After treatment, the cells were collected washed with ice‐cold Phosphate Buffered Saline (PBS), drained and added to ice‐cold RIPA buffer. The cells were lysed on ice for 20 min and centrifuged at 13,523 *g* at 4°C for 20 min, the supernatant was transferred to a fresh tube kept on ice. Protein G agarose beads were added to cell lysate and incubated at 4°C for 60 min to reduce the nonspecific binding of proteins. After centrifugation at 12,000 rpm at 4°C for 15 min, the supernatant was removal of to a fresh tube. Subsequently, primary antibody GSK‐3β was added and incubated overnight at 4°C. After that, protein G agarose beads were added and incubated with gentle rocking for 1–3 h at 4°C to capture the immune complex. Then, centrifuged for 1 min at 12,000 rpm at 4°C, and the pellet was washed with ice‐cold PBS and drained. The obtained pellet contained the protein G (antibody‐binding protein) was resuspended in SDS sample buffer, and heated to 95–100°C for 5 min, and then centrifuged for 1 min at 12,000 rpm. The obtained solution was then loaded on SDS‐PAGE gel and analysed by immunoblotting.

### 2.12 Mitochondria membrane potentials (MMP) measurement

Mito‐Tracker Red CMXRos (Beyotime Bio‐tech.) was used to evaluate MMP. According to the instructions, after TNF‐α inducement and TDZD‐8 treatment, cells were washed with PBS, stained with fluorescent probe for 30 min at 37°C in dark, replaced medium and observed under a fluorescence microscope (Olympus IX73, Olympus).

### 2.13 Mitochondrial reactive oxygen species assessment

Mitochondrial ROS was detected by MitoSOX Red mitochondrial superoxide indicator (Thermo Scientific) which is a novel fluorogenic dye for highly selective detection of superoxide in the mitochondria of live cells. Briefly, the cells were inoculated on a 24 well plate and induced with TNF‐α for 4 h and treatment with 1 μm TDZD‐8 for 24 h. Subsequently, the cells were incubated with 5 μm MitoSOX in the dark at 37°C for 10 min and detected under fluorescence microscope (Olympus IX73, Olympus).

### 2.14 Statistical analysis

All statistical analyses were conducted with SPSS 21.0 software package, and all data were evaluated by one‐way analysis of variance (one‐way anova) with repeated measures followed by Bonferroni post hoc tests and presented as mean ± SD. Data of PWT analysis were presented as mean ± SEM. Significance was described as *p* < 0.05.

## 3 RESULTS

### 3.1 Intratibial inoculation of breast cancer cells causes bone destruction and mechanical allodynia

**FIGURE 1:**
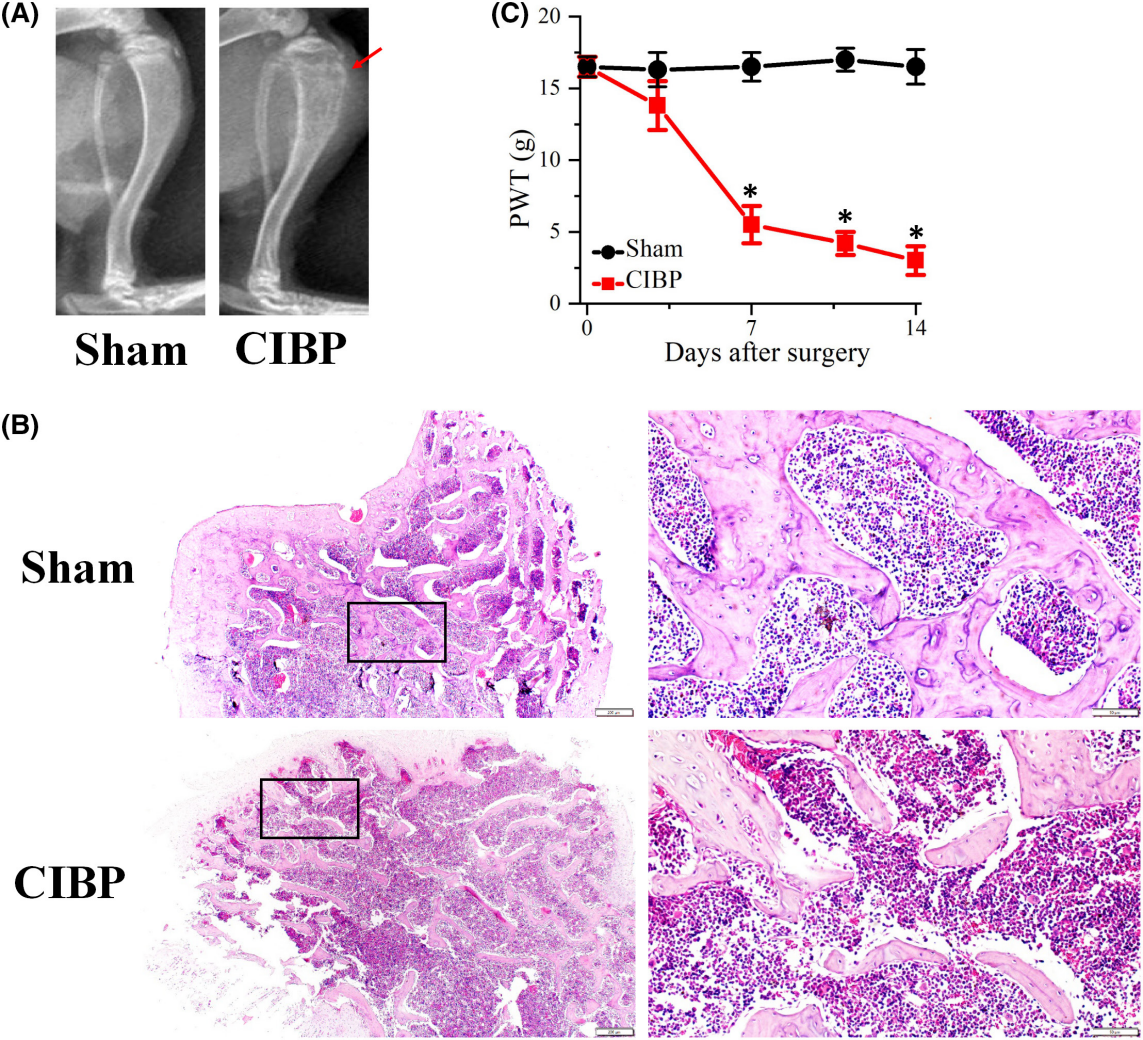
Bone destruction and mechanical allodynia caused by intratibial inoculation of breast cancer cells. (A) Representative radiograph of sham and cancer induced bone pain (CIBP) rats. Arrow indicates the position of bone destruction. (B) Haematoxylin and eosin staining examined the changes of trabecular bone of tibia. Scale bars: fist lane, 200 μm; second lane, 50 μm. (C) Comparison of paw withdrawal threshold (PWT) values between sham group and CIBP group. Data are expressed as the mean ± SEM (*n* = 6), *p < 0.05 vs. sham group. Data represent five independent experiments

CIBP rat model was established by intratibial inoculation of MRMT‐1 cells and validated using behavioural and morphological tests. X‐ray tibial radiographs exhibited a significant bone destruction in CIBP rats on post‐operation day (POD) 14 (Figure 1A), compared with sham group. H&E staining of tibia (bone sections) showed a deficiency of trabecular bone (Figure 1B). While no bone damage was observed in sham group. Mechanical PWT test was used to analyse the pain sensitivities. As shown in Figure 1C, PWT values of CIBP rats were significantly decreased at POD 7 (*p* < 0.05), POD 11 (*p* < 0.05) and POD 14 (*p* < 0.05). The result indicated that with the prolonged inoculation of tumour cells, the mechanical sensitivity of CIBP rats increased gradually. No significant PWT changes were detected in the sham rats.

### 3.2 Inoculation of cancer cells induces spinal inflammation

**FIGURE 2:**
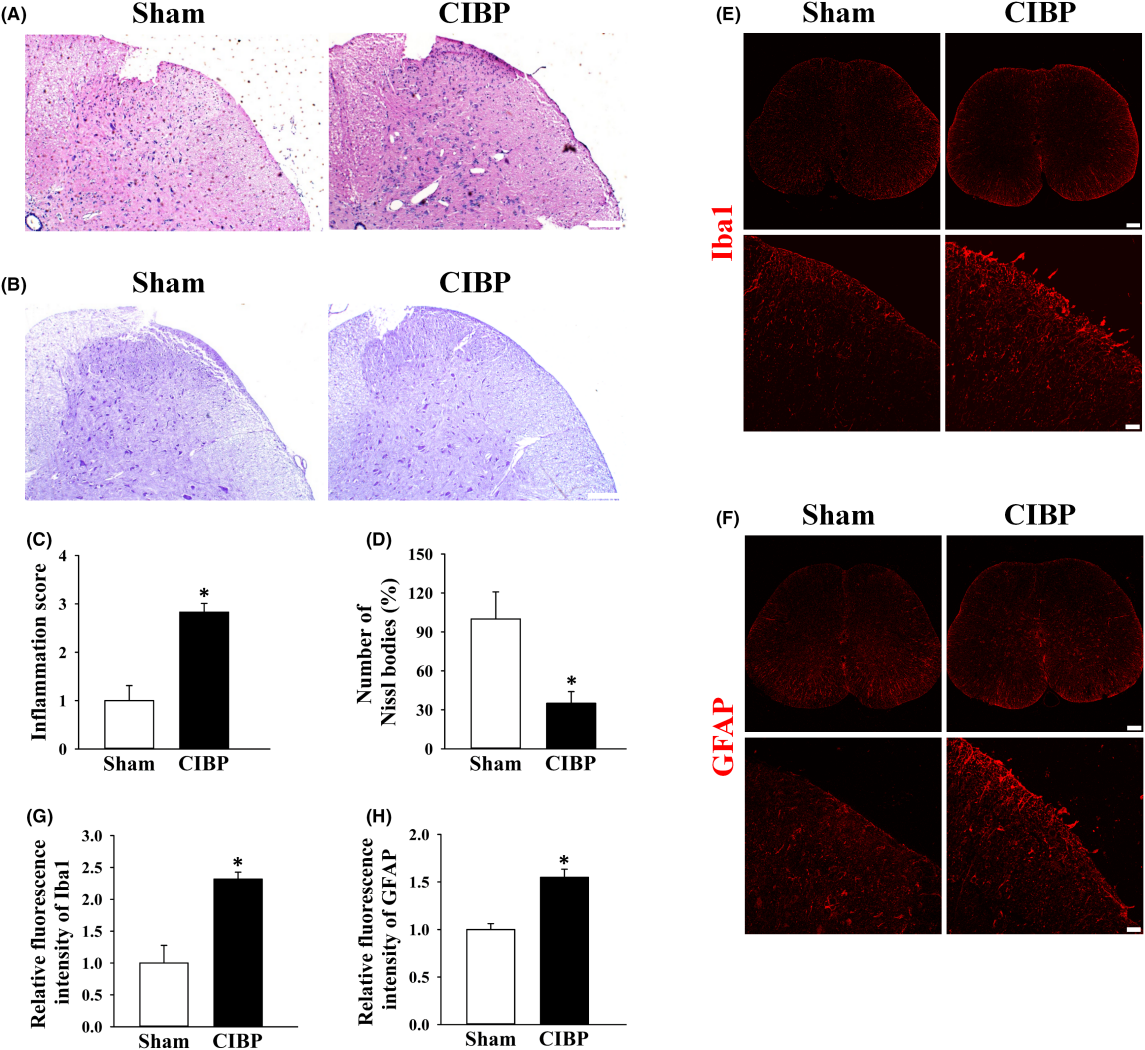
Spinal inflammatory response, number of Nissl bodies and activation of glial cells of sham and cancer induced bone pain (CIBP) rats. (A) Inflammatory cells infiltration of lumbar spinal dorsal horn from sham and CIBP rats were examined by Haematoxylin and eosin staining. Scale bar: 50 μm. (B) Nissl bodies in spinal dorsal horn from sham and CIBP rats were detected by Nissl staining. Scale bar: 50 μm. (C) Quantitative analysis of inflammation score in (A). *p < 0.05 vs. sham group. (D) Quantitative analysis of numbers of Nissl positive cells. *p < 0.05 vs. sham group. (E and F) Representative images of Iba1 and GFAP immunostaining on sections of spinal cords from sham and CIBP rats. (G and H) Quantitative analysis of Iba1 (E) and GFAP (F) fluorescence intensity in spinal dorsal horns from sham and CIBP rats. Scale bars: first line, 200 μm; second line, 20 μm. Data were showed as the mean ± SD. *p < 0.05 vs. sham group. Data represents five independent experiments

Histological morphology analysis showed severe infiltration of inflammatory cells in both grey matter and white matter, especially in grey matter, were observed in lumbar spinal dorsal horn of CIBP rats (Figure 2A). Relative inflammation score in CIBP rats was 2.83 ± 0.18 (*p* < 0.05 vs. sham group, Figure 2C). Nissl staining was used to detect Nissl bodies changes where was an important part for protein synthesis in neurons. When neuron was damaged, Nissl bodies would be significantly reduced. Our results showed that the number of Nissl bodies in grey matter of spinal dorsal horn of CIBP rats were significantly reduced (Figure 2B). Nissl positive cells in CIBP was 35 ± 9 (*p* < 0.05 vs. sham group, Figure 2D).

Inflammatory response mainly mediated by astrocyte and microglia in central nervous system. Compared with sham group, the fluorescence intensities of microglial marker Iba1 and astrocytic marker GFAP in spinal cord of CIBP rats were increased obviously (Figure 2E and Figure 2F). Relative fluorescence intensity of Iba1 and GFAP in CIBP group were 2.31 ± 0.11 (*p* < 0.05 vs. sham group, Figure 2G) and 1.54 ± 0.08 (*p* < 0.05 vs. sham group, Figure 2H), respectively. These data illustrated that activation of spinal glial and inflammatory response was triggered in CIBP rats.

### 3.3 Damaged spinal mitochondria in CIBP rats

**FIGURE 3:**
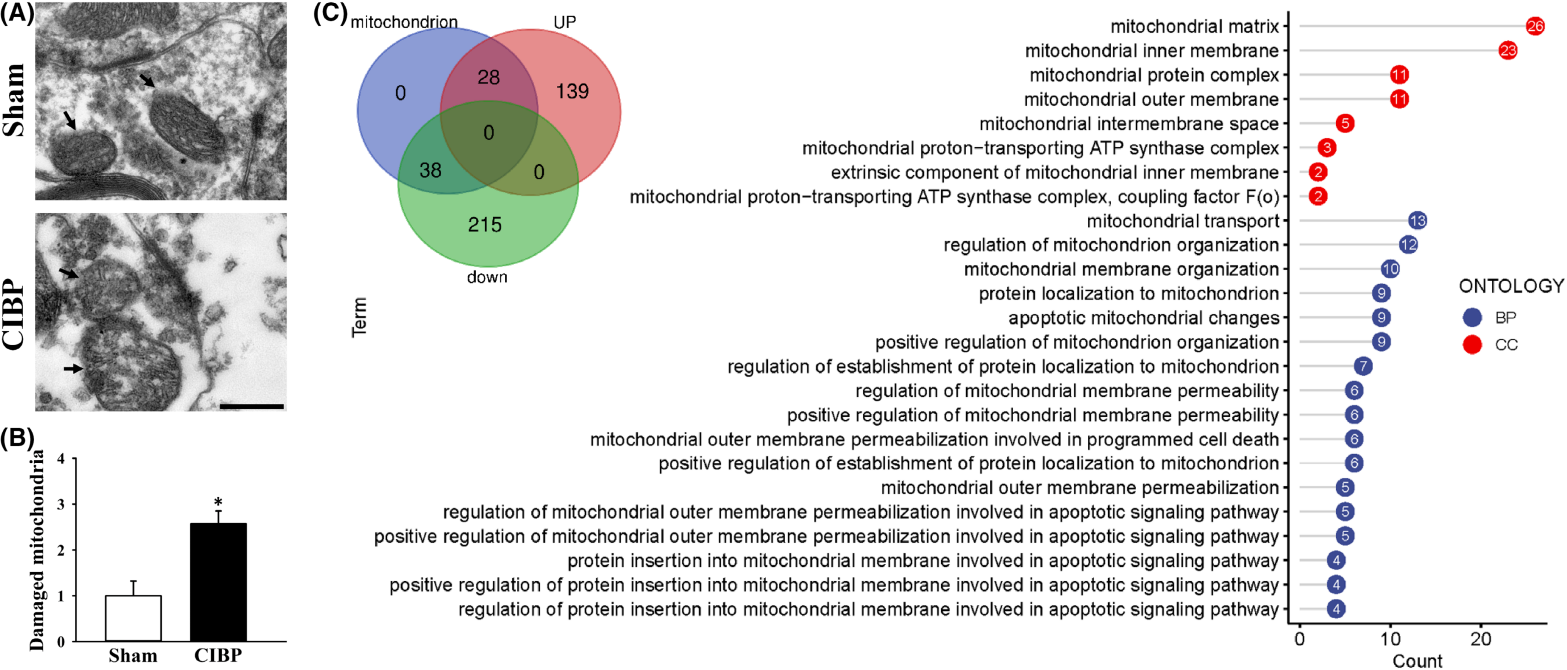
Changes of spinal mitochondrial morphology and functional proteins. (A) Spinal mitochondrial structure was observed by TEM. Representative TEM images of Sham and cancer induced bone pain (CIBP) rats. The arrows indicated mitochondria. Magnification: ×20,000. Scale bar: 500 nm. (B) Relative number of damaged mitochondria are showed as mean ± SD. *p < 0.05 vs. sham group. Data represent five independent experiments. (C) Gene Ontology analysis of mitochondrial related proteins identified by high‐throughput LC–MS/MS. Inner diagram shows the distribution of the identified proteins

Transmission electron microscopy was used to observe the morphological changes of spinal mitochondria. Mitochondria in CIBP group exhibited disrupted and discontinuous outer‐membrane and damaged cristae while mitochondrial structure in sham group exhibited whole compact outer‐membrane and normal cristae (Figure 3A). Relative ratio of damaged mitochondrial in CIBP group increased to 2.65 ± 0.32 (*p* < 0.05 vs. sham group, Figure 3B). To accurately quantify changes in mitochondrial proteome of CIBP rats, spinal cord proteins were analysed by LC–MS/MS. According to the Gene Ontology (GO) term (biological process (BP) and cellular component (CC)) analysis, 66 differentially expressed mitochondrial proteins between sham and CIBP rats were identified (Figure 3C). BP analysis showed changes in the protein levels of mitochondrial inner membrane, outer membrane, intermembrane space and ATP synthase complex. CC analysis revealed alternation of mitochondrial organization, mitochondrial membrane permeability and apoptotic signalling pathway. These data illustrated that bone metastasis of cancer cells induced mitochondrial damage and unmorally expression of related protein in spinal cord.

### 3.4 Inactivation of spinal GSK‐3β has an anti‐nociceptive effect on CIBP rats

**FIGURE 4:**
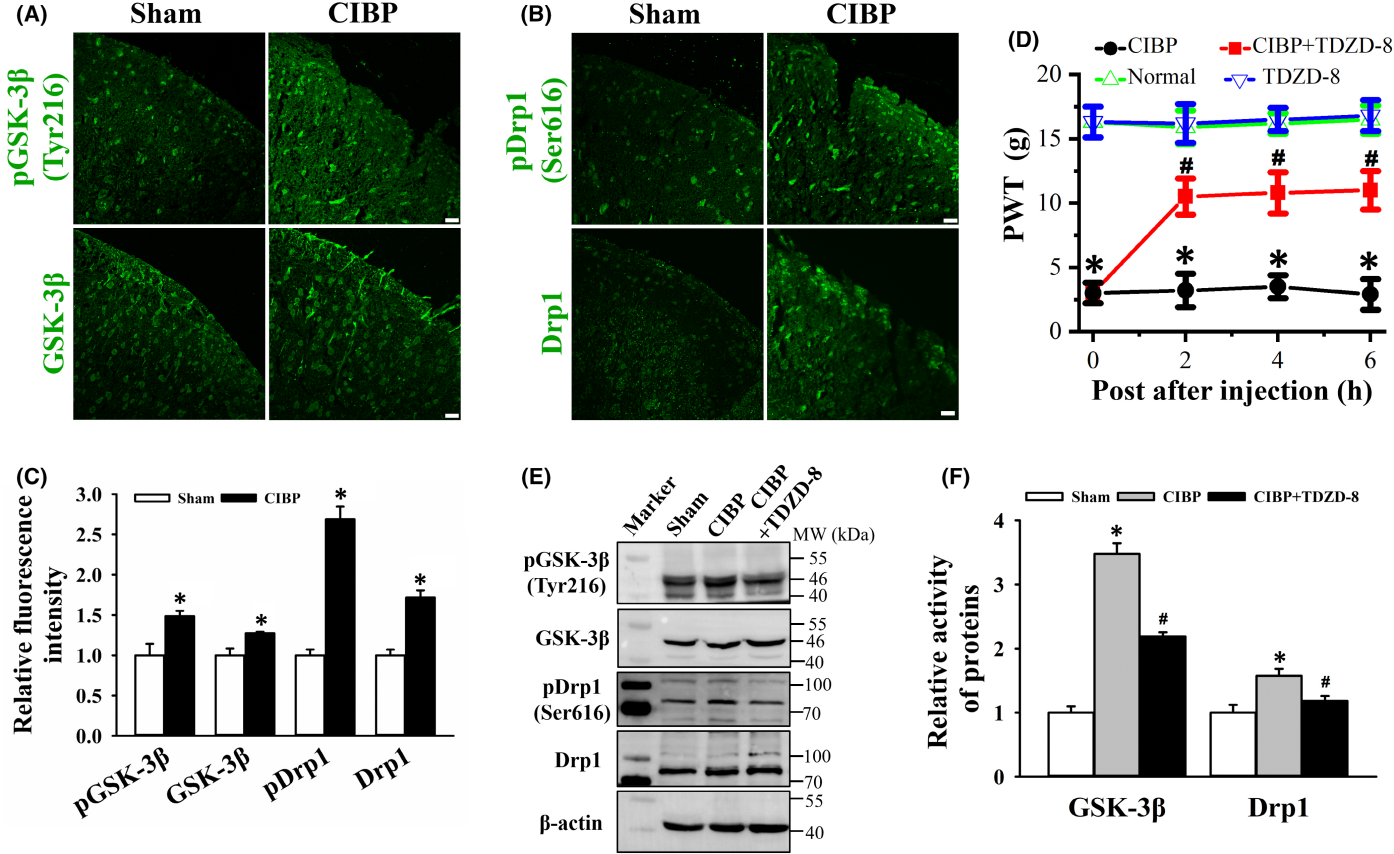
Effect of TDZD‐8 on spinal GSK‐3β and Drp1 activity, and mechanical pain behaviour of cancer induced bone pain (CIBP) rats. (A and B) Representative images of phosphorylated GSK‐3β (Tyr216), GSK‐3β, phosphorylated Drp1 (Ser616) and Drp1 immunofluorescence staining on spinal cord from sham and CIBP rats. Scale bars: 20 μm. (C) Quantitative analysis of phosphorylated GSK‐3β, GSK‐3β, phosphorylated Drp1 and Drp1 fluorescence intensity in the spinal cord. Data are expressed as the mean ± SD, *p < 0.05 vs. sham group. (D) Comparison of paw withdrawal threshold (PWT) values among normal, TDZD‐8, CIBP and CIBP + TDZD‐8 groups. All data are expressed as mean ± SEM (*n* = 6), *p < 0.05 vs. normal group. ^#^ *p* < 0.05 vs. CIBP group. (E) Western blot analysis of GSK‐3β, phosphorylated GSK‐3β at Tyr216, Drp1 and phosphorylated Drp1 at Ser616 in spinal cord of sham, CIBP and CIBP + TDZD‐8 rats. (F) Quantitative analysis of relative activity of GSK‐3β and Drp1, and are expressed as the ratio of pGSK‐3β/GSK‐3β and pDrp1/Drp1. Data are showed as mean ± SD. *p < 0.05 vs. sham group, ^#^ *p* < 0.05 vs. CIBP group

GSK‐3β is involved in the regulation of mitochondrial biogenesis, mitochondrial permeability and mitochondrial apoptosis and further contributes to inflammation and pain.^33^ Expression, activity and localization of GSK‐3β in spinal cord were detected by immunofluorescent staining. Since the kinase activity of GSK‐3β requires phosphorylation at Tyr216, we detected this site to represent GSK‐3β activity. The fluorescence intensity of phosphorylated GSK‐3β (Tyr216) and GSK‐3β in CIBP group was higher than that in sham group (Figure 4A). Relative intensity of phosphorylated‐GSK‐3β (Tyr216) and GSK‐3β was 1.48 ± 0.06 (*p* < 0.05 vs. sham group) and 1.27 ± 0.02 (*p* < 0.05 vs. sham group, Figure 4C). To determine the effect of GSK‐3β activity on CIBP rats' behaviour, TDZD‐8, a GSK‐3β inhibitor, was intrathecally injected into lumbar spinal cord. It was showed that TDZD‐8 treatment significantly decreased mechanical pain sensitivity of CIBP rats at 2, 4 and 6 h, presenting by increased PWT values. PWT values of normal and CIBP group at 2, 4 and 6 h after TDZD‐8 treatment were 3.2 ± 1.3 vs. 10.5 ± 1.4 (*p* < 0.05), 3.5 ± 0.9 vs. 10.8 ± 1.6 (*p* < 0.05) and 2.9 ± 1.2 vs. 11 ± 1.5 (*p* < 0.05), respectively (Figure 4D). TDZD‐8 treatment had no obvious effect on normal rats. Next, phosphorylation of GSK‐3β active site Tyr216 was analysed by immunoblot. In comparison with sham group, the level of phosphorylated GSK‐3β at Tyr216 was up‐regulated in CIBP group while TDZD‐8 treatment significantly reduced this up‐regulation (Figure 4E). As shown in Figure 4F, relative activity of GSK‐3β in CIBP group and CIBP+TDZD‐8 group was 3.48 ± 0.17 (*p* < 0.05 vs. sham group) and 2.19 ± 0.07 (*p* < 0.05 vs. CIBP group, Figure 4F), respectively. These data demonstrated that TDZD‐8 treatment inhibited spinal GSK‐3β activity and alleviated pain behaviours of CIBP rats.

### 3.5 TDZD‐8 treatment attenuates mitochondrial fission

GSK‐3β influences mitochondrial fission through regulating Drp1 phosphorylation,^34^ thus, we examined the expression and activity of Drp1 upon TDZD‐8 treatment. Immunofluorescent staining showed enhanced intensity of phosphorylated‐Drp1 (Ser616) and Drp1 in spinal dorsal horn in CIBP rats (Figure 4B). Relative fluorescence intensity of phosphorylated‐Drp1 (Ser616) and Drp1 in CIBP group was 2.69 ± 0.16 (*p* < 0.05 vs. sham group) and 1.72 ± 0.09 (*p* < 0.05 vs. sham group, Figure 4C). Change in Drp1 GTPase activity was determined by Western blot analysis of phosphorylated Drp1 at Ser616. Figure 4E showed that the activity of Drp1 GTPase was increased in spinal cord of CIBP rats, and TDZD‐8 treatment reduced Drp1 activity near to sham level. Relative activity of phosphorylated Drp1 at Ser616 in CIBP group and CIBP+TDZD‐8 group was 1.57 ± 0.11 (*p* < 0.05 vs. sham group) and 1.18 ± 0.08 (*p* < 0.05 vs. CIBP group, Figure 4F), respectively. These data indicated that increase in mitochondrial fission in CIBP rats was reduced after TDZD‐8 treatment.

### 3.6 TDZD‐8 treatment suppresses spinal NLRP3 inflammasome activation in CIBP rats

**FIGURE 5:**
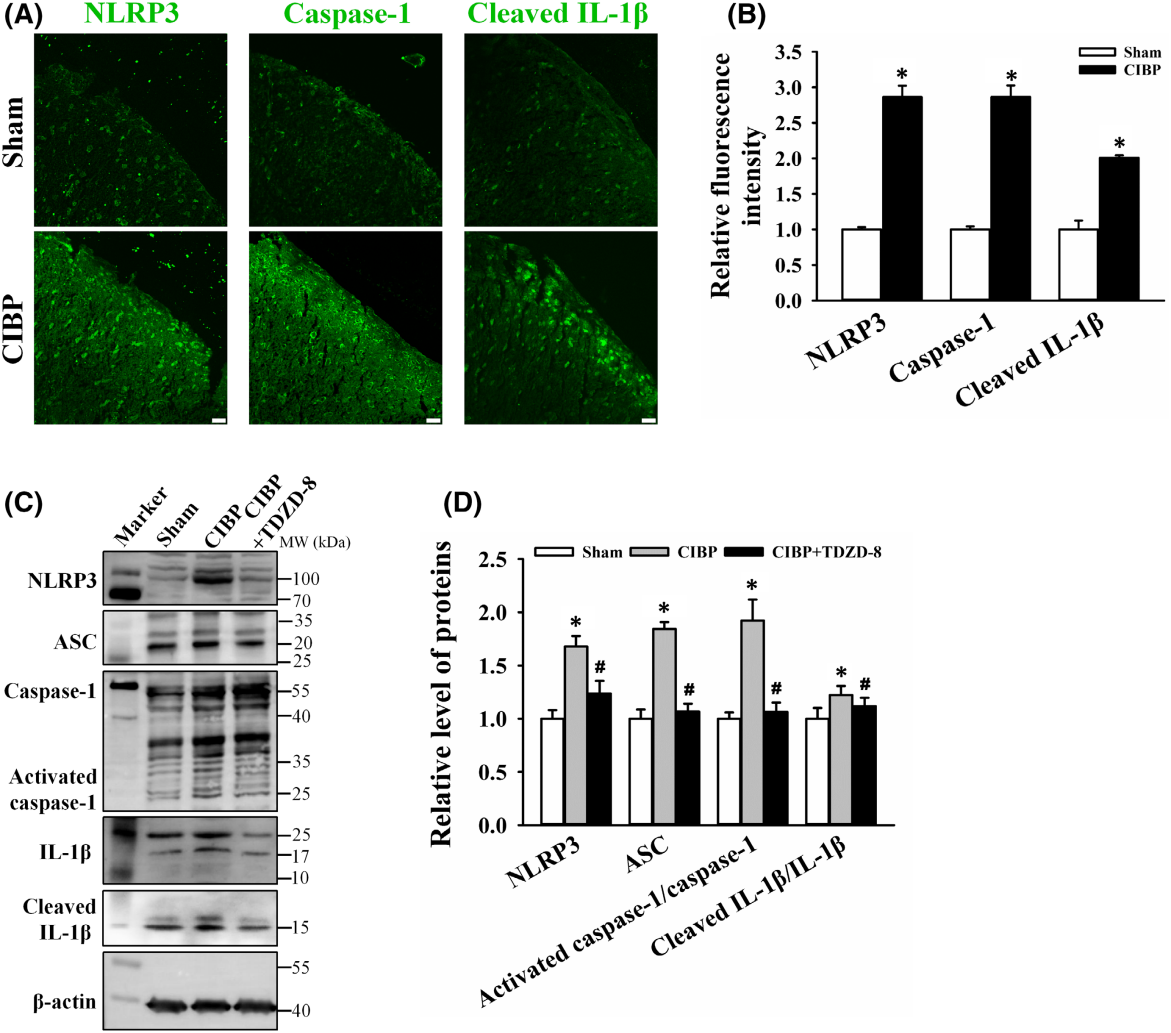
Effects of TDZD‐8 on spinal NLRP3 inflammasome cascade. (A) Representative images of NLRP3, caspase‐1 and cleaved IL‐1β immunofluorescence staining on spinal dorsal horns from sham and cancer induced bone pain (CIBP) rats. Scale bars: 20 μm. (B) Quantitative analysis of NLRP3, caspase‐1 and cleaved IL‐1β fluorescence intensities in spinal dorsal horns from sham and CIBP rats. Data are expressed as the mean ± SD. (C) Western blot analysis of NLRP3, ASC, caspase‐1, activated caspase‐1, IL‐1β and cleaved IL‐1β protein levels in spinal cord of sham, CIBP and CIBP+TDZD‐8 rats. (D) Relative levels of NLRP3, ASC, activated caspase‐1/caspase‐1 and cleaved IL‐1β/IL‐1β are shown as mean ± SD. *p < 0.05 vs. sham group, ^#^ *p* < 0.05 vs. CIBP group

Excessive mitochondrial ROS activated the nucleotide‐binding oligomerization domain (NOD)‐like receptor (NLR) family (NLRP3) inflammasome which induced the inflammatory response.^35^ NLRP3 inflammasome consists of NLRP3, the adapter apoptosis‐associated speck‐like protein containing a caspase recruitment domain (ASC) and the effector protease caspase‐1.^36^ Immunofluorescent staining showed that the fluorescence intensities of NLRP3, caspase‐1 and cleaved IL‐1β in spinal dorsal horns were increased in CIBP group (Figure 5A). Relative fluorescence intensity of NLRP3, caspase‐1 and cleaved IL‐1β in CIBP group were 2.86 ± 0.15 (*p* < 0.05 vs. sham group), 2.87 ± 0.16 (*p* < 0.05 vs. sham group) and 2.01 ± 0.04 (*p* < 0.05 vs. sham group, Figure 5B). Meanwhile, Western blot analysis showed that levels of NLRP3 inflammasome related proteins were all up‐regulated in spinal cord of CIBP rats (Figure 5C). Relative levels of NLRP3, ASC, activated caspase‐1/caspase‐1 and cleaved IL‐1β/IL‐1β were 1.68 ± 0.09, 1.84 ± 0.06, 1.92 ± 0.19 and 1.22 ± 0.08, respectively (*p* < 0.05 vs. sham group). TDZD‐8 treatment significantly reduced NLRP3 mediated inflammatory response. Relative levels of NLRP3, ASC, activated caspase‐1/caspase‐1 and cleaved IL‐1β/IL‐1β were reduced to 1.24 ± 0.12, 1.07 ± 0.07, 1.06 ± 0.09 and 1.11 ± 0.08, respectively (*p* < 0.05 vs. CIBP group, Figure 5D). These data suggested that NLRP3 mediated inflammatory response were activated and TDZD‐8 treatment suppressed spinal NLRP3 inflammasome in CIBP rats.

### 3.7 TDZD‐8 down‐regulates interaction of GSK‐3β and Drp1 and recovers mitochondrial function in C6 cells

**FIGURE 6:**
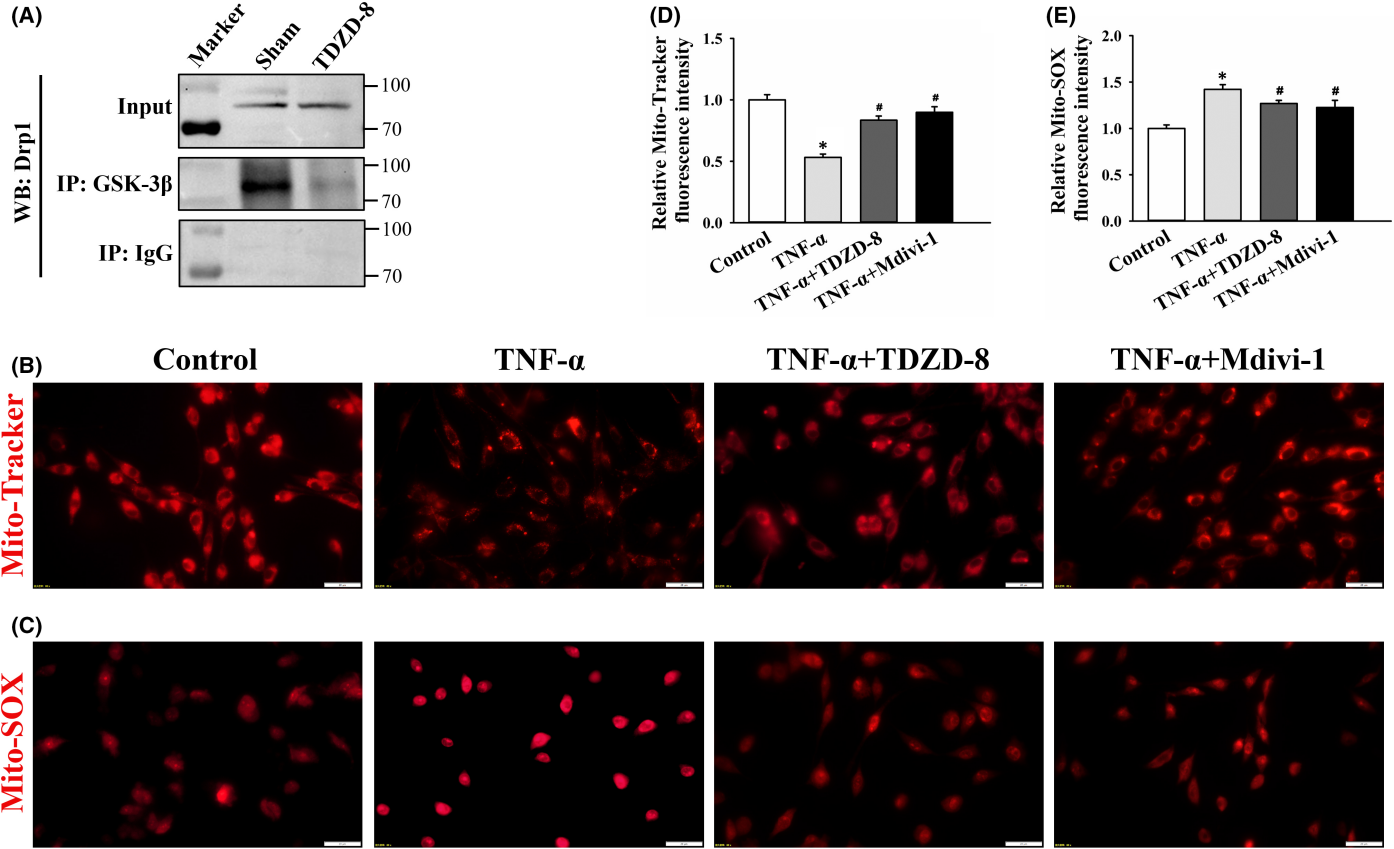
Effects of TDZD‐8 on the interaction of GSK‐3β and Drp1 and mitochondrial function in C6 cells. (A) Extracts from control and TDZD‐8 treated cells were immunoprecipitated with anti‐GSK‐3β antibody. The immunoprecipitation of Drp1 was detected by immunoblot with anti‐Drp1 antibody. Input represents the presence of the Drp1 protein prior to immunoprecipitation in the extracts. IgG represents the negative control. (B) Changes in mitochondrial membrane potential (MMP) of control, TNF‐α, TNF‐α + TDZD‐8 and TNF‐α + mdivi‐1 groups were detected by Mito‐Tracker analysis. Scale bar: 20 μm. (C) Mitochondrial ROS production in control, TNF‐α and TNF‐α + TDZD‐8 and TNF‐α + mdivi‐1 groups were indicated by the MitoSOX Red regent. Scale bar: 20 μm. (D and E) Quantitation of Mito‐Tracker Red CMXRos and MitoSOX Red fluorescence intensity from representative images. *p < 0.05 vs. control group, ^#^ *p* < 0.05 vs. TNF‐α group

To clarify the regulatory effect of GSK‐3β on Drp1, the immunoprecipitation was used to detect the interaction of GSK‐3β and Drp1. In Figure 6A, input demonstrated the existence of Drp1 in the cell extracts. The presence of Drp1 in the immunoprecipitates was detected by probing with anti‐Drp1 antibody. It showed that upon TDZD‐8 administration, the association of Drp1 and GSK‐3β was decreased, compared with control group. It was revealed that TDZD‐8 treatment reduced the interaction between GSK‐3β and Drp1.

To verify TDZD‐8 effect on mitochondrial function, changes in MMP and mitochondrial ROS levels under TDZD‐8 treatment were tested in C6 rat glioma cells. Changes in MMP were detected by Mito‐Tracker Red assay. While mdivi‐1 was a selective cell‐permeable inhibitor of Drp1 and used to protect MMP reducing. As shown in Figure 6B, TNF‐α induction resulted in significantly lower intensity with damaged MMP. TDZD‐8 and mdivi‐1 treatment increased mitochondrial intensity to the normal level. Relative fluorescence intensity of Mito‐Tracker Red in TNF‐α, TNF‐α + TDZD‐8 and TNF‐α + Mdivi‐1 were 0.53 ± 0.03 (*p* < 0.05 vs. control group), 0.83 ± 0.03 (*p* < 0.05 vs. TNF‐α group) and 0.90 ± 0.05 (*p* < 0.05 vs. TNF‐α group, Figure 6D), respectively. Effect of TDZD‐8 on mitochondrial ROS production was detected by MitoROS indicator. MitoSOX Red regent is live‐cell permeant and is rapidly and selectively targeted to the mitochondria. Once MitoSOX Red regent is oxidized by superoxide, it exhibits red fluorescence. As shown in Figure 6C, TNF‐α treatment increased the mitochondrial ROS while TDZD‐8 and mdivi‐1 treatment decreased the level of mitochondrial ROS induced by TNF‐α. The relative fluorescence intensity of MitoSOX Red regent in TNF‐α, TNF‐α + TDZD‐8 and TNF‐α + Mdivi‐1 groups was 1.42 ± 0.05 (*p* < 0.05 vs. control group) and 1.26 ± 0.03, and 1.22 ± 0.08 (*p* < 0.05 vs. TNF‐α group, Figure 6E), respectively. These data indicated that TDZD‐8 treatment significantly reversed TNF‐α induced MMP deficiency and high mitochondrial ROS level.

## 4 DISCUSSION

Bone as a preferential site for metastasis of breast and prostate cancers and approximately 70% of patients with advanced breast cancer develops bone metastasis.^37^ Cancer‐induced bone pain is one of the most prevalent and about 60%–84% of advanced cancer patients will experience different degrees of bone pain.^38^ Invasion of cancer cells in bone caused peripheral and central sensitization.^39^ In spinal cord, enhancement of synaptic excitatory and plasticity is accompanied by the activation of glial cells.^40^ In the current study, CIBP rats exhibited defective bone structure and increased mechanical hyperalgesia. Ultrastructure of spinal cord detecting by TEM showed increased the density and area of active zone and numbers of synapse vehicles around active zone in CIBP rats (Figure 3A). Local energy provided by mitochondria contributes the changes in morphology and structure of synapse.^41^, ^42^ Our proteomics data of spinal cord revealed that mitochondrial proteins related to morphology and function were changed (Figure 3C). Once overloaded, mitochondrial damage occurred.^43^ We also found synaptic damaged mitochondria in spinal cord of CIBP rats (Figure 3A,B). Taken together, abnormal morphology and structure of spinal synapses probably due to the dysregulation of mitochondrial proteins and consequently mitochondrial dysfunction.

GSK‐3β plays important roles in the modulating mitochondrial activity, dynamics and functions through several pathways.^44^ GSK‐3β can regulate mitochondrial biogenesis through phosphorylating peroxisome proliferators activated receptor gamma co‐activator 1 alpha (PGC‐1α).^45^ In addition, GSK‐3β also modulates mitochondrial motility in a tau protein dependent manner. Microtubule‐associated protein tau is a typical substrate of GSK‐3β.^46^ GSK‐3β causes abnormal mitochondrial fission by phosphorylating Drp1 at Ser616.^47^ Herein, increased GSK‐3β and Drp1 GTPase activity in spinal cord of CIBP rats were significantly inhibited by TDZD‐8 treatment (Figure 4). GSK‐3β participates in ATP and ROS production by regulating the activity of complex I, II, III and IV in mitochondrial respiratory chain. Moreover, GSK‐3β affects mitochondrial membrane potential by phosphorylating voltage‐dependent anion channel. Cell research indicated the inhibition of GSK‐3β attenuated TNF‐α‐induced low MMP and high mitochondrial ROS level (Figure 6). The results implied that GSK‐3β could be a potential target for ameliorating mitochondrial dynamics and function in CIBP rats.

Astrocytes and microglia undergo activation during CIBP. Induction of inflammatory response in glial cells was closely related to the pain behaviour of CIBP rats (Figures 2 and 5). Microglial NLRP3 inflammasome activation is emerging as a key contributor to neuroinflammation.^48^ NLRP3 inflammasome participates in the neuroinflammatory response through acting with ASC and caspase‐1.^49^ Activation of caspase‐1 consequently promotes the secretion of pro‐inflammatory cytokines, such as IL‐1β.^50^ Mitochondrial dysfunction and ROS generation are involved in the activation of NLRP3 inflammasome.^51^ Observation of damaged mitochondrial morphology in spinal cord, changes in the expression of mitochondrial morphology and function related protein, activation of NLRP3 inflammasome and increased expression of mature IL‐1β during cancer induced bone pain could be the inducement of hyperalgesia in the CIBP rats.

**FIGURE 7:**
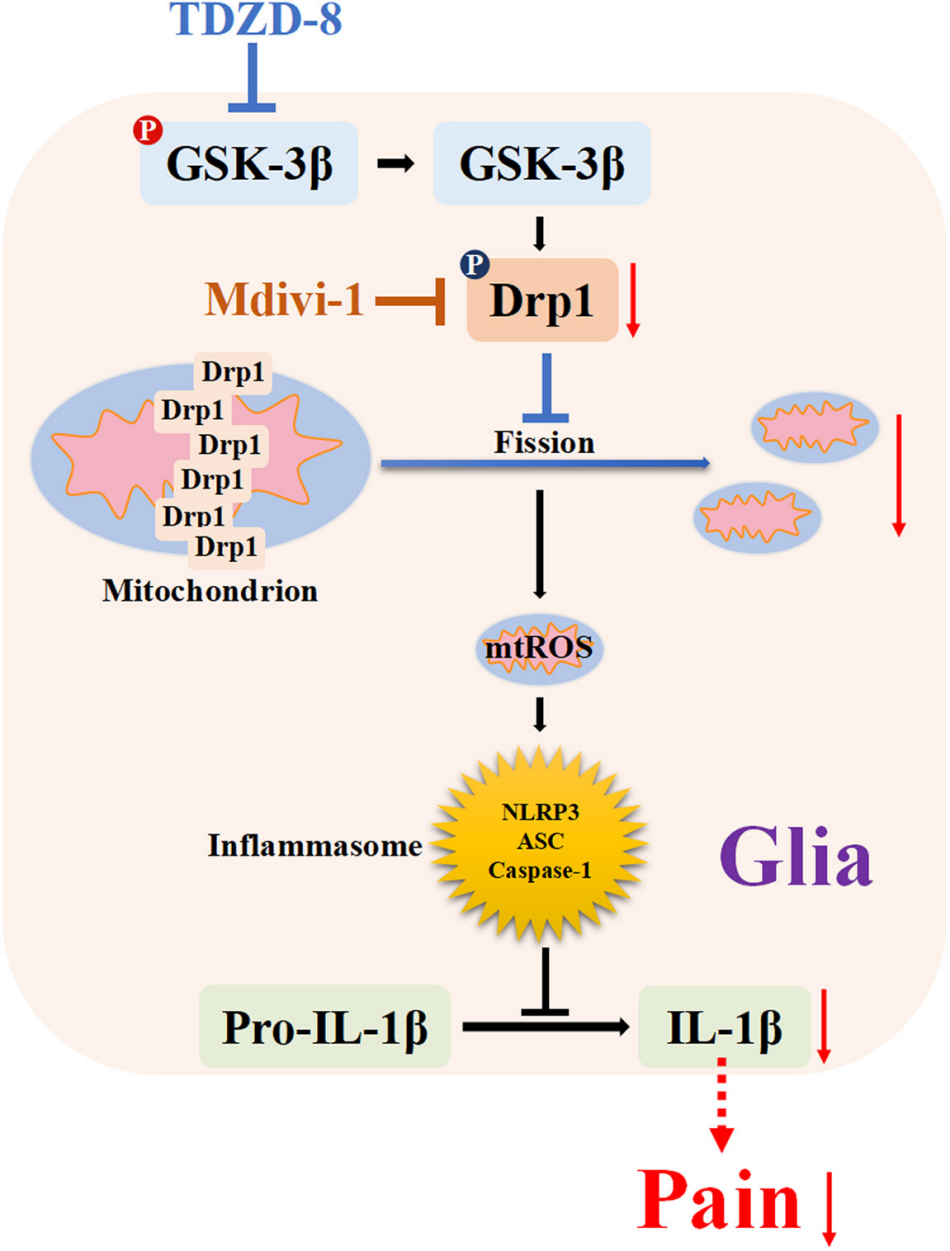
Schematic representation of the molecular mechanisms of GSK‐3β inhibition on pain behaviour of CIBP rats

In summary, metastasis of cancer cells in bone activates GSK‐3β/Drp1 pathway, resulting in mitochondrial fission and dysfunction, activating NLRP3 inflammasome and consequently inducing nociceptive response. Intrathecal injection of GSK‐3β inhibitor TDZD‐8 reduced Drp1 activity, maintained mitochondrial function, decreased NLRP3 inflammasome cascade and finally alleviated pain behaviour of CIBP rats (Figure 7).

## AUTHOR CONTRIBUTIONS

**He‐Yu Yang:** Data curation (equal); formal analysis (equal); methodology (equal); resources (equal); software (equal); supervision (equal); validation (equal); visualization (equal); writing – original draft (equal). **Feng Zhang:** Data curation (equal); investigation (equal); methodology (equal); project administration (equal); resources (equal); software (equal); supervision (equal); validation (equal); visualization (equal); writing – original draft (equal). **Meng‐Lin Cheng:** Investigation (equal); methodology (equal); project administration (equal); resources (equal); software (equal); supervision (equal); validation (equal); visualization (equal); writing – original draft (equal). **Ji Wu:** Formal analysis (equal); investigation (equal); methodology (equal); project administration (equal); resources (equal); software (equal); supervision (equal); validation (equal); visualization (equal); writing – original draft (equal). **Min Xie:** Resources (equal); software (equal); supervision (equal); validation (equal); visualization (equal). **Yu Liang‐Zhu:** Data curation (equal); formal analysis (equal); funding acquisition (equal); project administration (equal); writing – review and editing (equal). **Ling Liu:** Resources (equal); software (equal); supervision (equal); validation (equal); visualization (equal). **Jun Xiong:** Resources (equal); software (equal); supervision (equal); validation (equal); visualization (equal). **Haili Zhu:** Data curation (equal); formal analysis (equal); funding acquisition (equal); investigation (equal); methodology (equal); project administration (equal); resources (equal); software (equal); supervision (equal); validation (equal); visualization (equal); writing – original draft (equal).

## CONFLICT OF INTEREST

The authors confirm that there are no conflicts of interest.

## Data Availability

The data sets used and analyzed during the current study are available from the corresponding author on reasonable request.
